# Unusual Case of Takotsubo Cardiomyopathy Secondary to COVID-19 Vaccine: Case Report and Literature Review

**DOI:** 10.7759/cureus.25398

**Published:** 2022-05-27

**Authors:** Rafail Beshai, Jeffrey J Lee

**Affiliations:** 1 Internal Medicine, Jefferson Health New Jersey, Stratford, USA; 2 Cardiology, Cooper University Hospital, Camden, USA

**Keywords:** takotsubo, takotsubo cardiomyopathy, covid 19 vaccine complication, covid-19 vaccine, covid vaccine, corona virus, covid 19

## Abstract

COVID-19 is a serious disease with high morbidity and mortality around the globe. We present a case of a 45-year-old male who presents with substernal chest pain three days after receiving the second dose of his COVID-19 mRNA (Moderna) vaccine. A transthoracic echo showed reduced left ventricular ejection fraction of 25-30% with akinesis of the mid to distal anterior, anteroseptal, anterolateral, inferolateral, inferoseptal, and inferior walls. Patient symptoms improved significantly during his hospitalization. Repeat trans-thoracic echo four days after his hospitalization showed ejection fraction recovery without segmental wall motion abnormalities. This case demonstrates the importance of recognizing Takotsubo cardiomyopathy as a complication of COVID-19 vaccine.

## Introduction

The COVID-19 pandemic has placed an increased burden on healthcare resources around the globe [1]. It has caused more than five million deaths worldwide since its start in China in 2019 [2]. It has impacted the lives of millions more with devastating economic and social consequences [3]. Safe and effective prophylactic vaccines were urgently developed to contain the pandemic. Although COVID-19 vaccines are considered safe overall, publications about their side effects are still coming out. Clinicians should be aware of these unique side effects to aid with proper monitoring, treatment, and management.

## Case presentation

A 45-year-old male with no known past medical history presented with substernal chest discomfort while he was having dinner. The pain radiated to his jaw lasting approximately 2 hours. He never had this kind of pain before. He described the pain as a constant, severe stabbing pain that was progressively getting worse. Exertion exacerbated the pain. The pain was not pleuritic in nature. He was not a smoker and his family history is negative for any heart attack or stroke. He received his second dose of COVID-19 (Moderna) vaccine three days before coming to the hospital. He had had flu-like symptoms of fever, myalgia, and lethargy after his first dose. He was anxious to have similar symptoms after the second dose.

On admission, his blood pressure was 150/80. Remainder of his vitals were within normal limits. His labs were significant for high sensitivity troponin of 1700 ng/dl and brain natriuretic peptide (BNP) of 200 pg/ml. EKG showed ST-segment elevation in the anterior leads. Point of care ultrasound done by the ED physician showed left ventricular wall motion abnormality. A formal limited STAT transthoracic echo showed a reduced left ventricular ejection fraction of 25-30% with akinesis of the mid to distal anterior, anteroseptal, anterolateral, inferolateral, inferoseptal, and inferior walls (Video 1). ST-segment elevation myocardial infarction (STEMI) alert was called, and the patient was brought urgently to the cath lab. Urgent coronary angiography was performed and revealed no coronary stenosis. Computed tomography angiography (CTA) was negative for pulmonary embolism.

**Video 1:** Transthoracic echocardiogram (TTE) showing reduced left ventricular ejection fraction of 25-30% with akinesis of the mid to distal anterior, anteroseptal, anterolateral, inferolateral, inferoseptal, and inferior walls

The patient’s symptoms improved significantly during his hospitalization. Repeat transthoracic echo four days later showed recovery of the ejection fraction without segmental wall motion abnormalities (Video 2). EKG done before the patient's discharge showed normal sinus rhythm without ST-segment elevation. The patient was stable to be discharged home.

**Video 2:** A repeat transthoracic echocardiogram (TTE) four days after the patient's hospitalization showed recovery of the ejection fraction without segmental wall motion abnormalities

## Discussion

Acute myocardial infarction is the most clinically significant cause of chest pain, however, the differential diagnosis of chest pain is very broad. The differential diagnosis includes, but is not limited to, acute coronary syndrome, pericarditis, pulmonary embolism, and Takotsubo cardiomyopathy. Acute coronary syndrome was ruled out by cardiac catheterization that did not show any stenosis. Pericarditis and myopericarditis were unlikely as the inflammation markers were negative and there were no signs of pericardial effusion on the transthoracic echo. Pulmonary embolism as a cause of chest pain is unlikely in our case as the patient had a negative pulmonary CT angiogram. In this case, the diagnosis of Takotsubo cardiomyopathy was based on seven diagnostic criteria that were put forth by the European Society of Cardiology as our patient met all of those criteria [4].

Takotsubo cardiomyopathy (TCM) is still a medical mystery with a puzzling and complex presentation most likely similar to acute coronary syndrome with a largely unknown pathophysiology [4]. It was first recognized in Japan in 1990 [5]. The onset of stress cardiomyopathy is frequently triggered by intense emotional or physical stress. Around 30% of patients with TCM reported an emotional trigger such as panic, fear, and/or anxiety [6]. On the other hand, around 30% have no identifiable trigger [6]. The lowest mortality rates were in patients with an emotional trigger [7]. Recent publications have suggested an association between various coronavirus disease 2019 (COVID-19) vaccines and TCM (Table 1). An extensive review of the literature on PubMed and Google scholar was done and only a few case reports were found describing Takotsubo cardiomyopathy secondary to COVID-19 vaccines. To the best of our knowledge, we believe that this is the first case to describe TCM triggered by the second dose of the COVID-19 (Moderna) vaccine.

**Table 1:** Previous cases of Takotsubo cardiomyopathy (TCM) secondary to COVID-19 cases

Name of the author	Year	Age/gender	Type of Covid Vaccine	Which dose	Days after vaccine	Imaging findings	Time to normalization
Toida et al. [8]	2021	80 YO/F	Pfizer	1^st^	4 days	akinesia of the apical segments of the LV with EF of 48%	5 days
Fearon et al. [9]	2021	73YO/F	Moderna	1^st^	1 day	mid-ventricular ballooning of the LV, EF 20%	3 days
Jani et al. [10]	2021	65 YO/f	Moderna	1^st^	3 days	Hypokinesis of the mid and distal segments with EF of 35%	No repeat echo
Berto et al. [11]	2021	63 YO/F	Moderna	1^st^	1 day	mid-ventricular to apical ballooning with EF of 40%	No repeat Echo
Crane et al. [12]	2021	72 YO/M	AstraZeneca	1^st^	4 days	akinesis of the mid-distal left ventricular segments and severe hypokinesis of the apical cap with apical ballooning with EF of 37%-39%	days
Stewart et al. [13]	2022	50 YO/F	AstraZeneca	2nd	8 days	hypokinesis of the anterior and septal walls	No repeat echo before discharge

In this literature review, it was found that COVID-19 (Moderna) vaccine is responsible for 60% of Takotsubo cardiomyopathy cases secondary to any of the COVID-19 vaccines. The majority of cases happen after the first dose with the range of 1-4 days after. Our case was the first to report TCM triggered by the second dose of the COVID-19 (Moderna) vaccine. Similar to previous cases of Takotsubo cardiomyopathy, an echo done at the beginning of the admission is very important in order to have a baseline to track left ventricular recovery [14]. The time to recovery remains a mystery, as the exact left ventricular recovery mechanism is still unknown [14]. From our review, it was found that the time of normalization ranges from three to five days which is different from the average recovery time from other causes of Takotsubo cardiomyopathy where the median time to recovery of ejection fraction (EF) is 25 days [15]. The prevalence of TCM secondary to the COVID-19 vaccine is much more common in females, especially those who are post-menopausal. Our case was the second to describe a male case to develop TCM secondary to the COVID-19 vaccine.

Development of Takotsubo cardiomyopathy has been reported after the influenza vaccine in the past [16-18]. The pathophysiology behind vaccination-induced TCM is poorly understood but appears to be multifactorial. The etiology is most likely a combination of increased release of catecholamines or increased myocardial sensitization to them [19]. It has been shown cardiac sympathetic hyperactivity in patients with TCM using myocardial scintigraphy. Vaccinations in general cause a systemic inflammatory reaction, which can be appreciated by the increase in the body temperature after vaccinations [19]. These changes by the vaccination along with subclinical inflammation lead to an imbalance in the cardio-sympathetic system, with a sudden release of stress sympathetic hormones that have been demonstrated by the reduction in heart rate variability post-vaccination [19]. That is also how it is hypothesized that COVID-19 vaccines lead to TCM.

## Conclusions

Takotsubo cardiomyopathy is a rare disease with high morbidity and mortality. This case report is unique in the way that it presents TCM as a complication of the COVID-19 vaccine. Further research into the incidence and causes of TCM secondary to various COVID-19 vaccines should be done. This would decrease invasive procedures like cardiac catheterization and hence improve resource utilization overall. This research adds to the COVID-19 and COVID-19 vaccine database and will urge other researchers to look for similar symptoms and hence improving overall patient care. It is important for providers to be aware of this rare but potentially fatal side effect of the COVID-19 vaccine for better management of patients.
